# How Celluloid Is Made

**Published:** 1889-07

**Authors:** 


### HOW CELLULOID IS MADE.

Most celluloid is made in France. A roll of paper is slowly unwound, and at the same time is saturated with a mixture of five parts of sulphuric and two parts of nitric acid, which falls upon the paper in a fine spray. This changes the cellulose of the paper into pyoxylene (gun cotton). The excess of the acid having been expelled by pressure, the paper is washed with plenty of water, until all traces of acid have been removed. It is then reduced to a pulp, and passes on to a bleaching trough. It is this gun cotton which gives it its explosive nature. Most of the water having been gotten rid of by means of a strainer, it is mixed with from twenty to forty per cent. its weight in camphor, and a second mixing and grinding follows. This pulp is spread out in thin slabs, which are squeezed in a hydraulic press until they are as dry as chips. Then they are rolled in heated rollers, and come out in elastic sheets. They are from that point worked up into almost every conceivable form.

One can get celluloid collars, cuffs, hairpins, shirt fronts, cravats, pen holders, brushes, and combs, inkstands, knife handles, jewelry and everything else almost that you can imagine. In Paris, there is a room completely furnished with celluloid. The curtains, the door knobs, and even the matting were made of this material. To be sure, no matches were ever carried there. Indeed the room was never used. It was only a curiosity, and the man who owned it, owned the factory where it was made. These rooms will never be popular. Few men, even in this rapid age, care about being blown into the kingdom come in small fragments, scorched and shattered, and that would be the fate of the man who let a match fall in such a room.—*Universal Tinker.*

